# Genetics of Depression: Progress at Last

**DOI:** 10.1007/s11920-017-0803-9

**Published:** 2017-06-13

**Authors:** Niamh Mullins, Cathryn M. Lewis

**Affiliations:** 1 MRC Social, Genetic and Developmental Psychiatry Centre, Institute of Psychiatry, Psychology and Neuroscience, King’s College London, London, SE5 8AF UK; 2 Division of Genetics and Molecular Medicine, King’s College London, London, SE1 9RT UK

**Keywords:** Depression, Genetics, Genome-wide association study, Heterogeneity, Polygenic

## Abstract

**Purpose of Review:**

We will describe the success of recent genome-wide association studies that identify genetic variants associated with depression and outline the strategies used to reduce heterogeneity and increase sample size.

**Recent Findings:**

The CONVERGE consortium identified two genetic associations by focusing on a sample of Chinese women with recurrent severe depression. Three other loci have been found in Europeans by combining cohorts with clinical diagnosis and measures of depressive symptoms to increase sample size. 23andMe identified 15 loci associated with depression using self-report of clinical diagnosis in a study of over 300,000 individuals.

**Summary:**

The first genetic associations with depression have been identified, and this number is now expected to increase linearly with sample size, as seen in other polygenic disorders. These loci provide invaluable insights into the biology of depression and exciting opportunities to develop new biomarkers and therapeutic targets.

## Introduction

Major depressive disorder (MDD) is a common psychiatric illness and global public health problem [1]. It is the third leading cause of years lived with disability worldwide and a major contributor to early mortality from suicide [2]. Alleviating the burden of this costly disease is an important priority; however, limited understanding of the biological basis of depression has hindered the development of novel treatments and interventions.

Depression is a complex disorder with a heritability of 37% estimated from twin studies [3]. Despite robust evidence for a genetic component, identifying the specific genetic variants involved in the disorder has been a major challenge. Genome-wide association studies (GWAS) test differences in allele frequencies between disease and control groups at millions of common single nucleotide polymorphisms (SNPs) across the genome. These differences may be functionally relevant to the disease or may represent loci which are transmitted in linkage disequilibrium with a causative polymorphism. Early GWAS studies of MDD were not promising, despite having sample sizes similar to successful studies for other common diseases and traits, including psychiatric disorders. In a GWAS of over 9000 clinically ascertained MDD cases and 9000 healthy controls conducted by the Psychiatric Genomics Consortium (PGC), no SNPs reached the genome-wide significance threshold [4]. The CHARGE (Cohorts for Heart and Aging Research in Genomic Epidemiology) Consortium conducted a GWAS of depressive symptoms in over 30,000 individuals which also failed to identify any genetic associations [5].

## Challenges of Depression Genetics

There are several reasons why identifying risk loci for MDD has proven difficult. First, like most complex diseases, depression is a polygenic disorder arising from the combined effect of many genetic variants with individually small effect sizes [6]. Several sources provide evidence for the polygenic architecture of depression, despite a lack of genome-wide significant loci. Polygenic risk scoring uses association statistics from a discovery GWAS to weight the genotypes of individuals in an independent test sample and sums these effects across multiple SNPs into a polygenic risk score (PRS) [7]. Differences in PRS between cases and controls in the independent sample show that the PRS is capturing genetic susceptibility that is predictive of disease status. PRS for MDD generated from results of the PGC GWAS showed modest, although significant prediction for depression in independent samples (*R* ^2^ = 0.6%, *P* < 10^−6^), consistent with the presence of small genetic effects that the original GWAS was underpowered to detect at genome-wide significance [4]. SNP heritability (${h}_{\mathrm{SNP}}^2$ of MDD in the PGC GWAS was 0.21 (s.e. 0.021) [4, 9], again confirming the polygenic etiology of MDD. Large sample sizes are essential to detect small individual genetic effects, and pooling samples within research consortia has been key to the success of GWAS on many human traits.

The second characteristic of MDD which poses challenges to genetic analysis is its high lifetime prevalence of ~15% [1]. For a common disorder, the mean difference in phenotypic liability between case and control groups is smaller, for both unscreened and screened controls, and thus power to detect allele frequency differences between them is reduced. Power calculations show that samples 2.4-fold larger are needed for GWAS of MDD compared with schizophrenia (prevalence 1%), to identify a variant that explains the same proportion of risk [10]. Third, the heritability of MDD is modest, at 37%, compared with other psychiatric disorders, meaning that risk alleles are likely to have smaller effect sizes [3, 11]. To account for this lower heritability, samples 4–5 times larger would be required for MDD than schizophrenia to capture an equal amount of genetic variance [10].

Finally, depression is a particularly heterogeneous disorder. Some genetic heterogeneity is inherent to polygenicity; affected individuals may have different combinations of risk alleles and unaffected individuals will also carry many of these variants. But subphenotyping of the nine core symptoms of MDD indicates that almost 1500 symptom combinations can fulfill the diagnostic criteria and that two patients with a diagnosis of MDD may not have a single symptom in common [12]. Subtypes of depression such as recurrence or early-onset may be more heritable [3, 13]. Another striking example of heterogeneity is sex differences, with depression twice as prevalent among women than men and twin studies indicating that ~45% of the genetic liability to MDD is not shared between sexes [14–16]. Polygenic risk scoring methods also enable us to look for genetic similarities across traits and suggest that postpartum depression may be more genetically similar to bipolar disorder, that typical depression shows more pleiotropy with schizophrenia, and that atypical depression, characterized by increased appetite and weight, additionally shares genetic effects with BMI [17, 18]. These findings together provide compelling evidence that depression is likely composed of subtypes with differences in biological etiology and a heterogeneous genetic architecture. Therefore, the successful identification of genetic associations with MDD requires either increased sample sizes or empirically driven efforts to reduce heterogeneity. This review will outline recent genetic studies on depression which have adopted such strategies. Studies are described in detail, showing how each has advanced our understanding of the genetic underpinnings of depression, with summary information presented in Table 1.

**Table 1:** Recent genome-wide association studies on depression

Study	Year	Total N	Cases	Controls	Cohort	Ancestry	Depression phenotype	GWAS hits	Putative genes	*h* ^2^_SNP_ (s.e.)
PGC [4]	2013	18,759	9240	9519		European	Lifetime MDD established using structured clinical interviews	0	–	0.21 (0.021)^a^
CHARGE [5]	2013	34,549			34,549	European	Depressive symptoms in past weeks assessed by questionnaires	0	–	Not calculated
CONVERGE [20•]	2015	10,640	5303	5337		East Asian	Recurrent MDD in women	2	*SIRT1*, *LHPP*	0.21 (0.030)
SSGAC [24•]	2016	180,866	16,471	58,835	105,739	European	Depressive symptoms in past 2 weeks assessed by 2 questions; lifetime MDD	2	*KSR2*, *DCC*	0.04 (0.004)
23andMe [22•]	2016	307,354	121,380	338,101		European	Self-report of diagnosis or treatment for major depression	17	*TMEM161B-MEF2C*, *VRK2*, *L3MBTL2*, *NEGR1*, *RERE*, *HACE1-LIN28B*, *SORCS3*, *OLFM4*, *PAX5*, *MEIS2-TMCO5A*, *intergenic*, *RSRC1-MLF1*, *intergenic*, *SLC6A15*, *NEGR1*, *KIAA0020-RFX3*	0.06^b^
CHARGE + PGC [32•]	2016	70,017	9240	9519	51,258	European	Depressive symptoms in past weeks assessed by questionnaires; lifetime MDD	1	*FHIT*	0.30 (0.040)

*MDD* major depressive disorder

^a^On the liability scale, given a prevalence of 15%

^b^In the discovery cohort, on the liability scale, given a prevalence of 25%

## CONVERGE Consortium

The CONVERGE (**C**hina, **O**xford and **V**irginia Commonwealth University **E**xperimental **R**esearch on **G**enetic **E**pidemiology) Consortium has collected a large depression cohort with detailed clinical, genetic and environmental data that is a powerful resource to dissect the etiology of depression [19, 20•]. The study aimed to ascertain a more homogeneous sample by restricting the phenotype to recurrent severe depression in women. Using low-coverage sequencing of 5303 Han Chinese MDD cases and 5337 controls screened to exclude MDD, two SNPs on chromosome 10 showed evidence of association: one near the *SIRT1* gene and the other in an intron of *LHPP* [20•]. Both loci replicated in an independent Chinese sample and the genetic signal at the *SIRT1* locus increased when further restricting the sample to melancholia, a more severe subtype of MDD [20•]. This study demonstrates the value of focusing on a homogeneous phenotype where genetic effects should be larger and easier to detect, even at the expense of a smaller sample size. SIRT1 is involved in the biogenesis of mitochondria, which are the cell’s energy-producing organelles. Supporting the genetic association, the CONVERGE consortium report increased mitochondrial DNA in MDD cases versus controls, with the amount of increase positively correlated with stressors such as childhood sexual abuse and lifetime adverse events [21].

Although these genetic associations are a considerable step forward, the variants identified in individuals of East Asian ancestry have low frequencies in populations of European ancestry, and therefore no replication in the PGC depression samples or other studies has been achieved [20•, 22•]. The trans-ancestry genetic correlation between the PGC and CONVERGE GWAS results is ~0.3, indicating there are likely population differences in the genetic etiology of MDD, a finding with important implications for future studies [23]. Further comparison of the studies using genetic correlation and polygenic risk scoring weakly supports an overlap of SNP effects between the studies and strengthens when focusing on female only and recurrent MDD cases from the PGC [23]. This indicates that some of the genetic differences between the PGC and CONVERGE results may be due to differences in the specific MDD phenotype studied.

## Social Science Genetic Association Consortium

The Social Science Genetic Association Consortium (SSGAC) has pursued the alternate strategy of increasing sample size, by analyzing multiple cohorts with heterogeneous measures of depression [24•]. They utilized data from two case-control studies of MDD: summary statistics from the PGC GWAS (9240 MDD cases, 9519 healthy controls) and dbGaP-accessible genotypes from the GERA (Resource for Genetic Epidemiology Research on Adult Health and Aging) study (7231 MDD cases, 49,316 controls) [4, 25]. These clinical samples were meta-analyzed with a GWAS on a measure of depressive symptoms in the UK Biobank, where adults in the general population were asked two questions about feelings of unenthusiasm or disinterest and depression or hopelessness in the past 2 weeks [26]. Combining these datasets resulted in a sample of 180,866 individuals and found two genome-wide significant associations with “depressive symptoms” which replicated on look up in an independent depression GWAS by 23andMe [24•]. One SNP is in the *KSR2* (kinase suppressor of ras 2) gene and the other is in the *DCC* gene, which encodes a transmembrane receptor involved in axon guidance. The ${h}_{\mathrm{SNP}}^2$ for depressive symptoms from the total sample was 0.04 (s.e. 0.004), which is considerably lower than the estimates from clinically ascertained MDD samples (~0.2 in both the PGC and CONVERGE studies) [9, 27]. This may result from mixing heterogeneous measures of depression which are influenced by different combinations of genetic variants and the weak information on depression symptoms from just two questions. Nevertheless, the SSGAC attributes the success of their study to exploiting the genetic correlation between clinical depression and depressive symptoms to combine studies and increase sample size [24•]. While such a strategy may increase power for individual SNPs which influence both clinical depression and depressive symptoms, it may dilute associations for SNPs which only play a role in one phenotype and this has implications for replicating specific associations in different samples.

## 23andMe

The direct-to-consumer genetic testing company 23andMe (Mountain View, CA) also took the approach of increasing sample size. They used self-report data from consumers who participated in their research initiative and ascertained 75,607 individuals reporting previous clinical diagnosis or treatment for major depression and 231,747 individuals reporting no history of depression [22•]. They carried out meta-analysis of these results with the PGC GWAS results and then analyzed a replication sample of an additional 45,773 cases and 106,354 controls from 23andMe. A total of 17 independent SNPs from 15 regions reached genome-wide significance after joint analysis over all three data sets (Table 1) [22•]. Two of the loci were significant in both the meta-analysis and independent replication sample. In a locus spanning *MEF2C* (myocyte enhancer factor 2C) and *TMEM161B* (transmembrane protein 161B), two independent SNPs were significant. MEF2C is a transcription factor which plays a role in synaptic learning and memory and variants in the gene have been implicated in epilepsy, mental retardation, and schizophrenia [28–30]. The other locus encompasses the *NEGR1* gene, encoding neuronal growth regulator 1, which is involved in neurite outgrowth [31].

The strategy of less intensive phenotyping used in this study is a novel approach in psychiatric research, as cases have traditionally been ascertained using structured clinical interviews. To demonstrate the validity of the self-report measure, the authors calculated the genetic correlation between the results from the 23andMe study and those from the PGC GWAS. There was a high positive correlation of 0.72 (s.e. 0.09) between the results indicating common variant genetic overlap [22•]. However the ${h}_{\mathrm{SNP}}^2$ from the meta-analysis of the 23andMe discovery cohort and the PGC GWAS was 0.06, showing a substantially lower genetic component than the PGC ${h}_{\mathrm{SNP}}^2$ estimate of 0.21 [9, 22•]. This indicates that while the phenotypes are genetically correlated, the genetic signal in the 23andMe sample is likely weaker than in the PGC, which could reasonably be due to some diagnostic misclassification. The success of this 23andMe study in identifying genetic variants at genome-wide significance shows that large sample size can outweigh any reduction in power from additional heterogeneity or limited clinical information. Genotyping is now inexpensive compared with conducting detailed clinical interviews and 23andMe’s light-phenotyping approach may be more likely to attract the large number of participants required in the absence of high-quality phenotype information.

## CHARGE Consortium and PGC

Depression can be conceptualized along a spectrum of severity from subthreshold or minor depression to MDD of varying severity (e.g., mild, moderate, severe). Using a continuum approach to depression may augment statistical power because sample size can be increased substantially and individuals who fall anywhere along the phenotypic spectrum can be included. This was the rationale for combining the results of the CHARGE consortium GWAS of depressive symptoms and the PGC GWAS on MDD [32•]. Depressive symptoms were evaluated in individuals over 40 years old using validated questionnaires (mostly using the Center for Epidemiological Studies Depression Scale CES-D), which focused on depressive symptoms in the previous weeks rather than lifetime. This meta-analysis of a broad depression phenotype identified one genome-wide significant SNP, which replicated in an independent sample comprising newly ascertained MDD cases from the PGC and individuals assessed for depressive symptoms from the Health and Retirement Study [32•]. The SNP is located in an intron of *FHIT*, which is expressed in several brain regions and encodes a tumor suppressor protein also involved in oxidative stress and the circadian clock [32•].

In this study, the genetic correlation (*r*_g_) between depressive symptoms and MDD was 1.00 (s.e. 0.2) which supports the concept of a depression continuum capturing similar genetic underpinnings to a study of depression cases and controls. Notably the ${h}_{\mathrm{SNP}}^2$ of the broad depression phenotype was 0.3 (s.e. 0.04), which was greater than the ${h}_{\mathrm{SNP}}^2$ of depressive symptoms or MDD separately (0.04 (s.e. 0.01) and 0.21 (s.e. 0.02), respectively) [32•]. Testing the genetic correlation between different phenotypic measures before combining them can be informative about heritability in the subsequent sample and can be used to assess whether the sample size achieved will be sufficient to outweigh any heterogeneity introduced.

## Power and Study Design

The power of these studies to identify MDD-associated variants differs considerably by sample size and design. We calculated the genotype relative risk (GRR) which the study had 50% power to identify (Table 1), assuming a multiplicative model, allele frequency of 0.3, MDD prevalence of 15%, and fully screened controls [33]. The power of 50% was chosen to reflect the polygenic architecture of MDD, where many SNPs of modest effect sizes contribute, and each study has low power to detect a specific variant, but higher power to detect a subset of SNPs having a pre-specified GRR. Using standard power calculations, the 23andMe study would have 50% power to detect a variant with GRR 1.024, but the PGC MDD study could only detect a GRR of 1.11. However, such power calculations make simplistic assumptions about study design, for example that selected participants are divided into MDD cases and controls (defined as non-cases), with cases generally being over-sampled from the population. In practice, studies such as CONVERGE and some PGC MDD cohorts select severe, recurrent cases of MDD and exclude any individuals with mild to moderate depression. This selection of severe cases and healthier controls with no history of depression increases the power of the study by inducing a larger difference in allele frequency between cases and controls. In contrast, study power will be reduced by any misclassification of cases and controls, which may be more likely in studies based on self-report or limited phenotypic information at a single time point.

Two of the studies listed in Table 1 use a quantitative phenotype of the number of depressive symptoms (SSGAC, CHARGE). The CHARGE study of 51,258 participants would have 50% power to detect a variant accounting for 0.0058% of trait variance. A study of 180,000 participants, similar to SSGAC, could detect a variant accounting for 0.017% of trait variance (with 50% power), but the SSGAC study used only two questions on depressive symptoms, reducing its power from this theoretical value.

The studies described here illustrate two approaches to dissect the genetic contribution to depression: through a case-control study of lifetime diagnosis of depression or using a continuous measure of the count of depressive symptoms, usually covering the previous 2 weeks. Although the time scales for these measures differ, the genetic correlation between these measures is high, for example *r*_g_ = 1 between CHARGE and the PGC MDD study [32•]. The relationship between the power of a case-control and continuous phenotype was derived by Yang et al. [34] and shows that a cohort study with a continuous phenotype on *N* individuals has lower power than a case-control study with *N*/2 cases and *N*/2 controls when the disease prevalence is below 10%. This validates the design of studies such as CONVERGE, ascertaining recurrent cases of MDD where the population prevalence in China is already low at 3.6% [35]. In Western countries where MDD prevalence is 15–20%, studies based on an underlying quantitative trait may have higher power than an equivalently sized case-control study.

Studies must balance the trade-off between gains in power from increased sample size or reduced heterogeneity. As the results of CONVERGE and the 23andMe studies show, both approaches can be successful in identifying genetic variants for depression, and researchers need to decide which strategy maximizes the use of their resources. Since depression is a common disorder, large sample sizes can be accrued through consortia and inventive new methods such as leveraging electronic medical records, population biobanks, and online recruitment. One limitation of mixing heterogeneous measures of depression or less intensive phenotyping is that any associations discovered may be more difficult to interpret. But the approach of increasing sample size can be used to find loci whose role in MDD can then be dissected in follow-up samples with more detailed phenotypic data, even if these have smaller sample size. Large samples with different depression phenotypes will help to disentangle the genetic background of different forms of depression.

## Environment

While the focus of this review is on genetics, the role of the environment in depression cannot be ignored, with twin studies showing that it accounts for 63% of the variance [3]. In contrast to genetic associations, the environmental risk factors are well-established and include social isolation, unemployment, and relationship stressors [36]. Childhood abuse or neglect is one of the strongest environmental risk factors, more than doubling the risk for depression in adult life [37]. Gene-by-environment interactions (G×E) whereby genetic effects are moderated by specific environmental factors have long been postulated to play a role in depression. Most G×E research has focused on candidate genes such as the serotonin transporter promoter polymorphism (*5-HTTLPR*) interacting with stressful life events or childhood trauma. Over a decade’s worth of studies on this interaction has produced inconsistent results, and recently, an extensive, pre-registered meta-analysis concluded a lack of evidence for the *5-HTTLPR* interaction with environmental adversity [38•].

Since the genetic liability for depression is known to be polygenic, studies have begun to test for interactions between environmental factors and polygenic risk scores, which capture the cumulative effect of many common variants in a single measure. To date, two studies have reported no interaction between PRS for MDD and adult stressful life events in the etiology of depression [39, 40]. Two studies have found significant interactions between PRS for MDD and childhood trauma, albeit in opposing directions [39, 41]. The reason for these discrepant results is unclear but further research is warranted as the detection of G×E has implications for future research strategies to identify genetic associations. In the Netherlands Study of Depression and Anxiety (NESDA), PRS had a stronger effect on MDD in individuals exposed to childhood trauma, which suggests that focusing on exposed individuals could render genetic effects larger, more homogeneous and easier to detect [41]. However, in the RADIANT UK study, the effect of PRS on MDD risk was stronger in those unexposed to childhood trauma, suggesting that more power could be leveraged from GWAS by focusing only on individuals not exposed to trauma, as these MDD cases may have a stronger genetic predisposition. In summary, the analysis of cohorts with heterogeneous environmental exposures may also contribute to the difficulty in identifying genetic associations with MDD. Thus far, SNPs have been analyzed across average environmental backgrounds in GWAS but reducing environmental heterogeneity could be a valuable strategy to increase genetic effect sizes. There is a need for depression samples with good quality environmental data, which now can be more expensive and difficult to attain than genotype data.

## Conclusions

The first progress has been made towards identifying genetic variants involved in MDD with studies amassing the critical sample size necessary to reach an inflection point beyond which the number of genetic associations is expected to increase linearly with sample size [42•]. The critical goal of GWAS is to identify the biological pathways underpinning depression and even risk alleles with small effects could yield enormous insights. As sample sizes continue to increase, MDD GWAS will uncover more and more of the genetic architecture of this debilitating disorder, as we have seen in GWAS studies on schizophrenia [30]. The next challenge is to establish the molecular mechanisms by which GWAS loci mediate their effects and translate these into much-needed new biomarkers and therapeutic targets. We have turned the corner in identifying genetic variants for depression, and the next few years will bring exciting opportunities to turn biological findings into clinical tools.
